# *In Vitro* Study on the Antioxidant Potentials of the Leaves and Fruits of *Nauclea latifolia*

**DOI:** 10.1155/2014/437081

**Published:** 2014-06-11

**Authors:** Ademola O. Ayeleso, Oluwafemi O. Oguntibeju, Nicole L. Brooks

**Affiliations:** ^1^Oxidative Stress Research Centre, Department of Biomedical Sciences, Faculty of Health & Wellness Sciences, Cape Peninsula University of Technology, Bellville 7535, South Africa; ^2^Department of Wellness Sciences, Faculty of Health & Wellness Sciences, Cape Peninsula University of Technology, Cape Town 8000, South Africa

## Abstract

This study was carried out to investigate the *in vitro* antioxidant potentials of the leaves and fruits of *Nauclea latifolia*, a straggling shrub or small tree, native to tropical Africa and Asia. Hot water extracts of the leaves and fruits of *Nauclea latifolia* were assessed for their total polyphenolic, flavanol, and flavonol contents as well as 1-diphenyl-2-picrylhydrazyl (DPPH) scavenging ability, ferric reducing antioxidant power (FRAP), Trolox equivalence antioxidant capacity (TEAC), and oxygen radical absorbance capacity (ORAC) assays. The aqueous extract of the leaves was found to contain higher level of total polyphenols (11.63 ± 0.023 mg GAE/g), flavanol (1.45 ± 0.10 mg CE/g), and flavonol (2.22 ± 0.37 mg QE/g) than the extract of the fruits with values of 1.75 ± 0.02 mg GAE/g (total polyphenol), 0.15 ± 0.01 mg CE/g (flavanol), and 1.00 ± 0.13 mg QE/g (flavonol). Similarly, the aqueous extract of the leaves also exhibited higher DPPH (IC_50_ 20.64 mg/mL), FRAP (86.10 ± 3.46 **μ**mol AAE/g), TEAC (94.83 ± 3.57 **μ**mol TE/g), and ORAC (196.55 ± 0.073 **μ**mol TE/g) than the extract of the fruits with DPPH (IC_50_ 120.33 mg/mL), FRAP (12.23 ± 0.40 **μ**mol AAE/g), TEAC (12.48 ± 0.21 **μ**mol TE/g), and ORAC (58.88 ± 0.073 **μ**mol TE/g). The present study showed that *Nauclea latifolia* has strong antioxidant potentials with the leaves demonstrating higher *in vitro* antioxidant activities than the fruits.

## 1. Introduction

Free radicals, which belong to a group of reactive oxygen species (ROS), are produced through endogenous source, that is, the human body itself, and exogenous sources such as tobacco smoke, burning of fossil fuels, and ozone [1]. The imbalance between the production of ROS and the activity of the antioxidant defences is referred to as oxidative stress [1]. The inhibiting or preventive effects of herbs or spices against the deleterious consequences of oxidative stress are due to the presence of natural antioxidants in them [2]. Drug formulations that are antioxidant based are used in the prevention and treatment of complex diseases which include atherosclerosis, stroke, diabetes, Alzheimer's disease, and cancer [2, 3].

*Nauclea latifolia *Smith (family: Rubiaceae) is a straggling, evergreen, multistemmed shrub or small tree native to tropical Africa and Asia. It normally produces interesting flowers, edible, but not appealing, large red ball fruits with long projecting stamens [4]. Commonly used parts of* Nauclea latifolia* include the leaves, roots, stem, and fruits. The fruits serve as key source of food for the baboons, livestock, reptiles, birds, and man [4]. Phytochemicals majorly found in* Nauclea latifolia* include indole-quinolizidine, alkaloids (glycoalkaloids), and saponins [5]. Nkafamiya et al. [6] also reported that the fruits of* Nauclea latifolia* contain copper, iron, cobalt, calcium, magnesium, zinc, phosphorus, and vitamins (A, B_1_, B_2_, C, and E).

Throughout recorded history,* Nauclea latifolia *herbal remedies have been frequently seen in various places and are being used as major means of therapeutic medical treatment [7]. Traditionally, the infusions and decoctions from the stem bark and leaves of the plant are used for the treatment of malaria, stomach ache, fever, diarrhea, and nematodes infections [8–10]. Several studies have confirmed the health potentials of* Nauclea latifolia. *Some of its medicinal effects include antimalarial [11], antidiabetic [8], antihypertensive [12–14], antipyretic and antinociceptive [15], and anti-inflammatory and analgesic activities [7]. It is also known to have a strong antibacterial property [16, 17]. This study was conducted to investigate the antioxidant potentials in the aqueous extracts of the leaves and fruits of* Nauclea latifolia* as these could be the contributing factors to their health beneficial effects.

## 2. Materials and Methods

### 2.1. Preparation of Plant Extracts

The plant was identified and authenticated by a plant scientist (Mr Omotayo) and deposited at the herbarium (E-herbarium UHAE 261) in the Department of Plant Science, Ekiti State University, Ado Ekiti, Nigeria. The dried and powdered leaves and fruits (150 g) were extracted with 750 mL of hot (100°C) distilled water. After 30 min, the extracts were filtered out and used for the assays.

### 2.2. Liquid Chromatography-Mass Spectrometry (LC-MS) Technique

LC-MS was performed on a Dionex HPLC system (Dionex Softron, Germering, Germany) equipped with a binary solvent manager and autosampler coupled to a Brucker ESI Q-TOF mass spectrometer (Bruker Daltonik GmbH, Germany). Constituents of the aqueous plant extracts were separated by reversed phase chromatography on a Thermo Fisher Scientific C18 column 5 *μ*m, 4.6 × 150 mm (Bellefonte, USA), using a linear gradient of 0.1% formic acid in water (solvent A) and acetonitrile (solvent B) as solvent at a flow rate of 0.8 mL min^−1^, an injection volume of 10 *μ*L, and an oven temperature of 30°C. MS spectra were acquired in negative mode. Electrospray voltage was set to +3500 V. Dry gas flow was set to 9 L min^−1^ with a temperature of 300°C and nebulizer gas pressure was set to 35 psi.

### 2.3. Determination of Total Polyphenol, Flavanol, and Flavonol Contents

The total polyphenol content in the aqueous plant extracts was determined using the Folin-Ciocalteu's phenol reagent according to the method described by Singleton et al. [18] and was determined spectrophotometrically using a microplate reader and expressed as mg gallic acid standard equivalents (GAE) per gram sample. The flavanol content of the aqueous plant extracts was determined colorimetrically at 640 nm using the aldehyde DMACA and expressed as mg catechin standard equivalents (CE) per gram sample [19, 20]. The flavonol content of the aqueous plant extracts was determined spectrophotometrically at 360 nm and expressed as mg quercetin standard equivalents (QE) per gram sample [21]. All determinations were done in triplicates.

### 2.4. DPPH Free Radical Scavenging Activity

DPPH free radical scavenging activity of the aqueous plant extracts was carried out according to a method described by Zheleva-Dimitrova [22] with slight modifications. Briefly, 10 *μ*L of the different concentrations of the aqueous plant extracts was reacted with 190 *μ*L of DPPH solution (0.00625 g DPPH in 50 mL methanol) and the absorbance of the samples was determined after 30 min using a Multiskan Spectrum plate reader (Thermo Fisher Scientific, USA) at 517 nm. Free radical scavenging activity of the samples was expressed according to the equation below:

(1) Percent (%) inhibition of DPPH activity    =A0−AA0×100,

where *A* ^0^ is the absorbance of DPPH• in solution without an antioxidant and *A* is the absorbance of DPPH• in the presence of an antioxidant. IC_50_ value (concentration of sample where absorbance of DPPH decreases 50% with respect to absorbance of blank) of the sample was determined. All determinations were done in triplicates.

### 2.5. Ferric Reducing Antioxidant Power Assay

The FRAP assay was done using the method described by Benzie and Strain [23]. Briefly, 10 *μ*L of the diluted aqueous plant extracts was mixed with 300 *μ*L FRAP reagent in a 96-well clear plate. The FRAP reagent was a mixture (10 : 1 : 1, v/v/v) of acetate buffer (300 mM, pH 3.6), tripyridyl triazine (TPTZ) (10 mM in 40 mM HCl), and FeCl_3_·6H_2_O (20 mM). After incubation at room temperature for 30 min, the plate was read at a wavelength of 593 nm in a Multiskan Spectrum plate reader (Thermo Fisher Scientific, USA). Ascorbic acid (AA) was used as the standard and the results were expressed as **μ**mol AAE/g sample. All determinations were done in triplicates.

### 2.6. Trolox Equivalence Antioxidant Capacity Assay

The TEAC assay was carried out using the principle of 2,2′-azino-bis(3-ethylbenzothiazoline-6-sulphonic acid) (ABTS) radical scavenging activity according to a method described by Ou et al. [24]. ABTS^+^ solution was prepared a day before use by mixing ABTS salt (8 mM) with potassium persulfate (3 mM) and then storing the solution in the dark until the assay could be performed. The ABTS^+^ solution was further diluted with distilled water. Twenty five microlitres (25 *μ*L) of the diluted aqueous plant extracts was mixed with 300 *μ*L ABTS^+^ solution in a 96-well clear microplate. The plate was read after 30 min incubation at room temperature in a Multiskan Spectrum plate reader (Thermo Fisher Scientific, USA) at 734 nm. Trolox was used as the standard and results were expressed as *μ*mol TE/g sample. All determinations were done in triplicates.

### 2.7. Oxygen Radical Absorbance Capacity (ORAC) Assay

The ORAC assay was conducted according to the method of Re et al. [25] on a 96-well microplate using a Fluorescence plate reader (Thermo Fisher Scientific, Waltham, Mass., USA). The reaction consisted of 12 *μ*L of diluted aqueous plant extracts and 138 *μ*L of fluorescein (14 *μ*M), which was used as a target for free radical attack. The reaction was initiated by the addition of 50 *μ*L AAPH (768 *μ*M) and the fluorescence was (emission 538 nm, excitation 485 nm) recorded every 1 min for 2 hours. Trolox was used as the standard and results were expressed as *μ*mol/g sample. All determinations were done in triplicates.

### 2.8. Statistical Analysis

Results are expressed as the means ± standard deviations. The significance of difference between mean values of different plant extracts was determined by* t*-test using Graph Pad PRISM 5.

## 3. Results and Discussion

Essential source of new chemical substances with potential therapeutic effects is thought to be obtained from medicinal plants [7, 26, 27]. The antioxidant contents of medicinal plants may contribute to protection against diseases [28]. Natural antioxidants have attracted a great deal of public and scientific attention because of their health-promoting effects [29]. An imbalance between the production of reactive oxygen species (ROS) and the activity of the antioxidant defences leads to oxidative stress [1]. In the pathology of several human diseases such as atherosclerosis, inflammation, cancer, rheumatoid arthritis, and neurodegenerative diseases like Alzheimer's disease and multiple sclerosis, ROS have been implicated [1]. Attention is on antioxidant agents of natural origin due to their abilities to scavenge free radicals [28, 30]. Antioxidant capacity is associated with compounds that can protect a biological system against the damaging effect of ROS and reactive nitrogen species (RNS) [31].

### 3.1. Polyphenolic Contents in the Aqueous Extracts of the Leaves and Fruits of* Nauclea latifolia*

**Figure 1:**
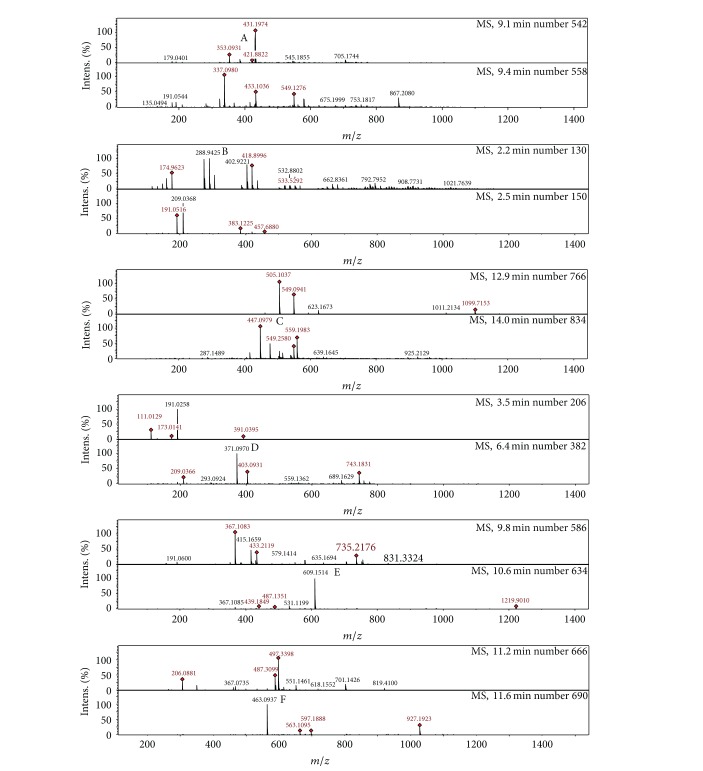
LCMS chromatogram of phenolic acid and flavonoids in the aqueous extract of* Nauclea latifolia* leaves. (A) Chlorogenic acid, (B) catechin, (C) cynaroside, (D) tangeritin, (E) rutin/hesperidin, and (F) hyperoside.

**Figure 2:**
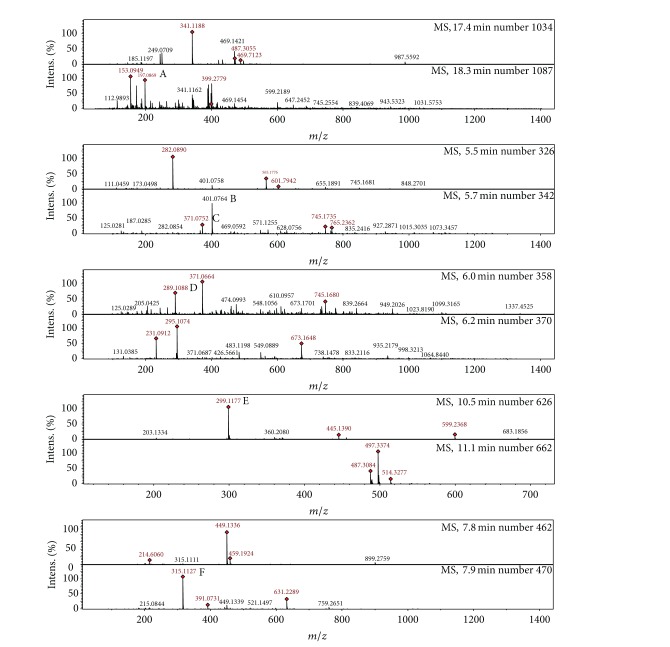
LCMS chromatogram of phenolic acid and flavonoids in the aqueous extract of* Nauclea latifolia* fruits. (A) Syringic acid, (B) nobiletin, (C) tangeritin, (D) catechin, (E) chrysoeriol, and (F) isorhamnetin.

**Figure 3:**
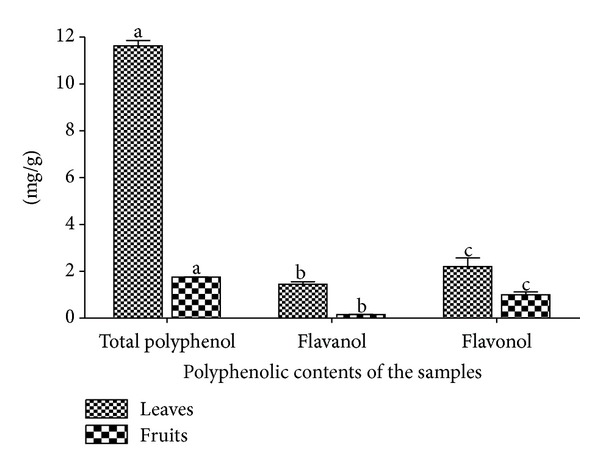
Total polyphenol, flavanol, and flavonol contents in the aqueous extracts of the leaves and fruits of* Nauclea latifolia. *All significant differences are at *P* < 0.05. ^a-a, b-b, c-c^Bars having the same alphabets are significantly different from each other.

Phenolic compounds are found usually in both edible and nonedible plants with several biological effects which include antioxidant activity [32].  Figure 1 shows that the aqueous extract of the leaves of* Nauclea latifolia* contains chlorogenic acid, catechin, cynaroside, tangeritin, rutin/hesperidin, and hyperoside.  Figure 2 shows that the aqueous extract of the fruits of* Nauclea latifolia* contains syringic acid, nobiletin, tangeritin, catechin, chrysoeriol, and isorhamnetin. In this study, the total polyphenolic contents of the aqueous plant extracts of the leaves and fruits were determined using the diluted Folin-Ciocalteu reagent. The total polyphenolic content was higher in the aqueous extract of the leaves than in the extract of the fruits with mean values of 11.63 ± 0.23 mg GAE/g and 1.75 mg ± 0.02 mg GAE/g sample, respectively (Figure 3). The flavanol content was also higher in the aqueous extract of the leaves (1.45 ± 0.10 mg CE/g) than in the extract of the fruits (0.15 ± 0.01 mg CE/g). In a similar vein, flavonol content was significantly higher in aqueous extract of the leaves (2.22 ± 0.37 mg QE/g) than in the extract of fruits (1.00 mg ± 0.13 mg QE/g) (Figure 3). Overall, the results showed that aqueous extracts of both the leaves and the fruits are rich in polyphenols with the leaves having higher polyphenols than the fruits.

### 3.2. DPPH Radical Scavenging Activity in the Aqueous Extracts of the Leaves and Fruits of* Nauclea latifolia*

**Figure 4:**
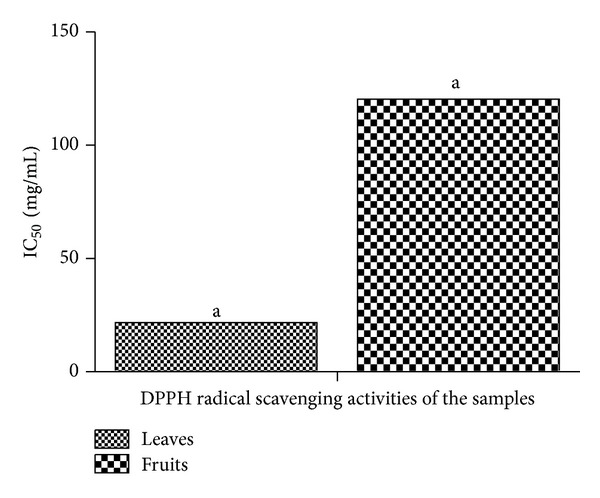
DPPH radical scavenging activity in the aqueous extracts of the leaves and fruits of* Nauclea latifolia. *All significant differences are at *P* < 0.05. ^a-a^Bars having the same alphabets are significantly different from each other.

DPPH radical is used as a stable free radical to determine the antioxidant activity of natural compounds and the scavenging of stable radical (DPPH) is considered a valid and easy assay to evaluate scavenging activity of antioxidants [33–35]. In this assay, purple color of DPPH is reduced to *α*,*α*-diphenyl-*β*-picrylhydrazine (yellow colored) when neutralised. The extent of the change in color is proportional to the concentration and strength of the antioxidants [28]. In this study, the aqueous extracts of both the leaves and the fruits were able to show free radical scavenging abilities (Figure 4). It was observed that the aqueous extract of the leaves had higher DPPH activity than the extract of the fruits with the concentration of sample where absorbance of DPPH decreases 50% with respect to absorbance of blank (IC_50_) to be 20.64 mg/mL and 120.33 mg/mL, respectively. The effect of antioxidants on DPPH could be due to their hydrogen donating ability [36].

### 3.3. Ferric Reducing Antioxidant Power in the Aqueous Extracts of the Leaves and Fruits of* Nauclea latifolia*

**Figure 5:**
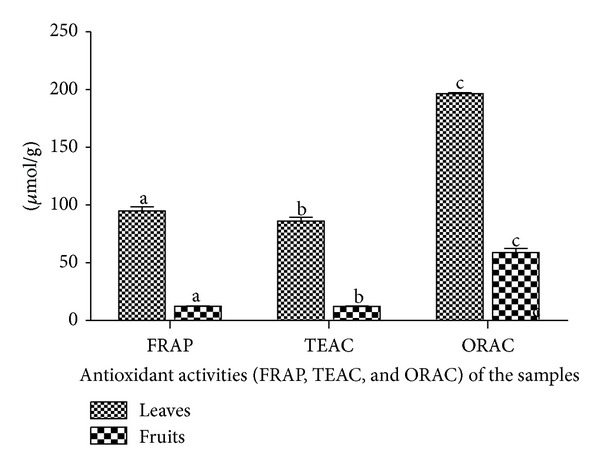
Antioxidant activities (FRAP, TEAC, and ORAC) in the aqueous extracts of the leaves and fruits of* Nauclea latifolia. *All significant differences are at *P* < 0.05. ^a-a, b-b, c-c^Bars having the same alphabets are significantly different from each other.

The extent at which the aqueous extract of the leaves and fruits of* Nauclea latifolia* could reduce ferric ions was done with FRAP assay. This assay is made possible by low molecular weight antioxidants of hydrophilic and/or hydrophobic nature [37]. FRAP assay has been used to compare antioxidant activity in plants and mammals [38–40]. The action of electron donating antioxidants causes a change in the absorbance at 593 nm due to the formation of blue colored Fe^+2^ tripyridyl triazine (TPTZ) compound from the colorless oxidized Fe^+3^ form [41, 42]. The aqueous extract of the leaves showed higher FRAP (86.10 ± 3.46 *μ*mol AAE/g sample) than the extract of the fruits (12.23 ± 0.40 *μ*mol AAE/g sample). It was clearly shown that even though the aqueous extracts of both the leaves and the fruits have the abilities to reduce ferric ions, the activity of the leaves was higher (Figure 5).

### 3.4. Trolox Equivalence Antioxidant Capacity in the Aqueous Extracts of the Leaves and Fruits of* Nauclea latifolia*

The TEAC assay is based on the suppression of the absorbance of radical cations of 2,2-azino-bis(3-ethylbenzothiazoline-6-sulfonate) (ABTS) by antioxidants in the sample when ABTS incubates with a peroxidase (metmyoglobin) and H_2_O_2_ [43, 44]. It entails a technique that produces a blue/green ABTS^+^ chromophore through the reaction of ABTS and potassium persulfate [30]. The assay is particularly interesting in plant extracts because the wavelength absorption at 734 nm eliminates color interference [45]. The results also showed a higher TEAC in the aqueous extract of the leaves than in the extract of the fruits with values of 94.83 ± 3.57 *μ*mol TE/g sample and 12.48 ± 0.21 *μ*mol TE/g sample, respectively (Figure 5). In this study, the antioxidant content of the aqueous plant extracts captured the free radical (ABTS) and resulted in a color loss and consequent reduction in absorbance which corresponds to the antioxidant concentration.

### 3.5. Oxygen Radical Absorbance Capacity in the Aqueous Extracts of the Leaves and Fruits of* Nauclea latifolia*

The ORAC assay is used to assess the antioxidant activity of biological substrates which ranges from pure compounds, that is, melatonin and flavonoids, to complex matrices such as vegetables and animal tissues [46]. It measures antioxidant inhibition of peroxyl-radical-induced oxidations and shows the radical chain breaking antioxidant activity by H-atom transfer [24, 31]. The ORAC assay uses 2,2′-azobis(2-amidinopropane) dihydrochloride (AAPH) for free radical generation. AAPH which is water soluble has been widely used as a free radical initiator for biological studies and the haemolysis caused by AAPH allows studies involving membrane damage induced by free radicals [47–50]. The results showed the mean value of ORAC for aqueous extract of the leaves to be 196.55 ± 0.073 *μ*mol TE/g sample while that of the fruits was 55.88 ± 0.073 *μ*mol TE/g sample (Figure 5). The ORAC results also showed the potency of the aqueous plant extracts to protect against oxidative damage.

In conclusion, the results from this study revealed that the aqueous extracts of both the leaves and the fruits of* Nauclea latifolia* have antioxidant potentials with the leaves demonstrating stronger activities. The medicinal properties of this plant could be due to its antioxidant potentials as evident from this present work. More studies are however needed for investigating its usefulness in the management and treatment of various diseases.
